# Condición biopsicosocial con enfoque de género de mujeres privadas de la libertad

**DOI:** 10.15446/rsap.V25n2.101923

**Published:** 2023-03-01

**Authors:** Kendy Madero-Zambrano, Carolyn Osorio-Madero, Shirley Fernández-Aragón, Zorayda Barrios-Puerta, Moraima Del Toro-Rubio, Sandra L. Vallejo-Arias

**Affiliations:** 1 KM: Enf. M. Sc. Auditoría y Sistemas de Calidad en Servicio de Salud. Corporación Universitaria Rafael Núñez. Cartagena, Colombia. kendy.madero@curnvirtual.edu.co Corporación Universitaria Rafael Núñez Auditoría y Sistemas de Calidad en Servicio de Salud Corporación Universitaria Rafael Núñez Cartagena Colombia kendy.madero@curnvirtual.edu.co; 2 CO: Psicol. Esp. Psicología Clínica. Corporación Universitara Rafael Nuñez. Barranquilla, Colombia. carolyn.osorio@curnvirtual.edu.co Corporación Universitaria Rafael Núñez Psicología Clínica Corporación Universitara Rafael Nuñez Barranquilla Colombia carolyn.osorio@curnvirtual.edu.co; 3 SF: Enf. M. Sc. Enfermería. Corporación Universitaria Rafael Núñez. Cartagena, Colombia. shirley.fernandez@curnvirtual.edu.co Corporación Universitaria Rafael Núñez Enfermería Corporación Universitaria Rafael Núñez Cartagena Colombia shirley.fernandez@curnvirtual.edu.co; 4 ZB: Enf. M. Sc. Educación. Corporación Universitaria Rafael Núñez. Cartagena, Colombia. zorayda.barrios@curnvirtual.edu.co Corporación Universitaria Rafael Núñez Educación Corporación Universitaria Rafael Núñez Cartagena Colombia zorayda.barrios@curnvirtual.edu.co; 5 MDT: Enf. M. Sc. Educación. Corporación Universitaria Rafael Núñez. Cartagena, Colombia. moraima.deltoro@curnvirtual.edu.co Corporación Universitaria Rafael Núñez Educación Corporación Universitaria Rafael Núñez Cartagena Colombia moraima.deltoro@curnvirtual.edu.co; 6 SV: Enf. M. Sc. Educación. Corporación Universitaria Rafael Núñez. Cartagena, Colombia. sandra.vallejo@curnvirtual.edu.co Corporación Universitaria Rafael Núñez Educación Corporación Universitaria Rafael Núñez Cartagena Colombia sandra.vallejo@curnvirtual.edu.co

**Keywords:** Prisioneras, características de la población, estado de salud, salud mental, resocialización, identidad de género *(fuente: DeCS, BIREME)*, Population characteristics, health status, mental health, resocialization, gender identity *(source: MeSH, NLM)*

## Abstract

**Objetivo:**

Identificar las condiciones biopsicosociales con enfoque de género de mujeres privadas de la libertad de la Cárcel Distrital de Cartagena (Colombia), en el año 2020.

**Materiales y Métodos:**

Estudio cuantitativo, descriptivo de corte transversal. La muestra estuvo conformada por 130 mujeres privadas de la libertad en la Cárcel Distrital de Cartagena; se utilizó un muestreo por conveniencia. Se aplicó un cuestionario tipo Likert que evaluaba los aspectos sociodemográficos, el estado de salud percibido y las condiciones de reclusión. La información se analizó mediante el *software* SPSS versión 27.

**Resultados:**

Las reclusas reportaron tener una edad entre 30 y 39 años (28,5%), así como tener hijos (91,5%), ser de estrato uno (74,6%) y ser bachilleres (53,8%). Entre los aspectos físicos se encontró: IMC normal (40%), no se reportaron ETS (96,2%), el estado de salud percibido fue bueno (48,5%). Con referencia a las condiciones ambientales, se presentaron pocas molestias (41,5%), estrés a menudo (50%) y relaciones interpersonales excelentes (48,5%).

**Discusión:**

A diferencia de otros estudios, los hallazgos hechos por la investigación muestran un panorama distinto en relación con las condiciones biopsicosociales de un grupo de mujeres privadas de la libertad, especialmente por los resultados de enfermedades, consumo de alcohol, drogas y tabaco. Sin embargo, es necesario desarrollar acciones para prevenir alteraciones cardiovasculares y de salud sexual y reproductiva. Por otro lado, se encontró la percepción de buen estado de salud y pocas molestias (comida, violencia, compañeras, etc.), así como buen ambiente interpersonal con los guardias y con el personal de la cárcel. Finalmente, se halló similitud con algunos estudios que evidencian estrés como consecuencia del quebranto de las relaciones familiares y las características propias de la reclusión.

## Abstract (en)

**Objective:**

To identify the biopsychosocial conditions with a gender approach of women deprived of liberty in the District Prison of Cartagena-Colombia 2020.

**Materials and Methods:**

Quantitative, descriptive, cross-sectional study. The sample consisted of 130 women deprived of liberty in the Cartagena District Jail, using convenience sampling. A questionnaire was applied to assess sociodemographic aspects, perceived health status and conditions of confinement. The information was analyzed using SPSS software version 27.

**Results:**

The inmates reported being 30-39 years old (28.5%), having children (91.5%), being from stratum one (74.6%), and having a high school diploma (53.8%). Among the physical aspects of the women, a normal BMI was highlighted (40%), no STD reported (96.2%), health status perceived as good (48.5%). Environmental conditions, few discomforts (41.5%), stress often (50%) and excellent interpersonal relations (48.5%).

**Discussion:**

Unlike other studies, the findings obtained by the research show a different panorama in relation to the biopsychosocial conditions of a group of women deprived of liberty; especially for the results of diseases, consumption of alcohol, drugs and tobacco; however, it is necessary to develop actions to prevent cardiovascular alterations and sexual and reproductive health. On the other hand, the perception of good health and few discomforts (food, violence, companions, etc.) was found, as well as a good interpersonal environment with prison guards and staff. Finally, similarities were found with some studies that show stress as a result of the breakdown of family relationships and the characteristics of imprisonment.

Uno de los castigos más severos que pueden ser impuestos a una persona lo constituye la privación de la libertad en una cárcel, esta implica perder por un tiempo el ejercicio de algunos derechos esenciales para el ser humano, y se convierte en sí misma en un factor de vulnerabilidad. Estos derechos corresponden a "la libertad de tránsito, el derecho a votar y ser votado, el derecho al trabajo, el derecho a la libertad de asociación, entre otros. También significa una restricción enorme a derechos como el de libertad de expresión, derechos de reunión y derecho a la privacidad", e inclusive, se transgrede el derecho a la salud 1,2.

La situación de encarcelamiento se piensa como algo más grave cuando se trata de mujeres, pues en la sociedad este grupo de población suele desempeñar la mayor parte del trabajo doméstico y atender responsabilidades familiares como el cuidado de los hijos (sobre todo si son muy pequeños), de personas dependientes o discapacitadas, entre otros. Así, "las mujeres se sienten doblemente castigadas ya que, por una parte, se genera sentimiento de culpabilidad" 3, como también afectaciones que se manifiestan por medio de la impotencia y la obsesión, al ser conscientes de la situación familiar y no poder hacer nada desde la prisión 4.

Adicionalmente, estas mujeres deben convivir con aspectos incómodos como requisas sobre los cuerpos desnudos, pérdida de la intimidad, además del aislamiento, los traslados constantes como forma de "infligir castigo, controlar, modular o cortar los flujos comunicacionales de las personas detenidas entre sí y con sus redes familiares", contexto que hace aún más críticas las condiciones en las cuales se encuentra esta población 5.

Como consecuencia, de las características propias de la cárcel, las reclusas se ven obligadas a convivir de modo permanentemente entre ellas, dado que no es fácil hallar espacios ni momentos propios o personales para la tranquilidad, el sosiego y la reflexión, y la convivencia forzada genera tensión y alerta recurrente, puesto que en la cotidianidad están presentes delincuentes cuya peligrosidad no se conoce bien. Estas condiciones desencadenan afectaciones tales como el aumento del nivel de estrés, que reduce el funcionamiento psicológico y repercute en un mayor nivel de desgaste físico y mental 6.

Es necesario poner el énfasis sobre la salud mental, habida cuenta de que es uno de los aspectos que más se ven afectados en las reclusas. De acuerdo con lo que se muestra en el trabajo de Aristizábal *et al.*^7^, la mitad de estas mujeres presentan trastornos depresivos, sin contar aspectos como los sentimientos de culpa, de inferioridad, la falta de confianza en sí mismas, así como los comportamientos relacionados con la evitación y el aislamiento. Sobre esta misma línea se encuentran García y Ordoñez 8, quienes sustentan que en algunas ocasiones las reclusas pierden el contacto con sus familiares y amigos, y ello se relaciona con su bienestar físico y emocional.

Es necesario mencionar que un gran número de las reclusas que se encuentran en los centros penitenciarios son madres. En este sentido, Martínez 9 enuncia en su estudio que estas mujeres no solo padecen sufrimiento al estar lejos de sus hijos, sino que se sienten culpables por la separación. Muchas de las reclusas pierden toda relación con sus hijos durante su tiempo de condena, de manera que no sienten el apoyo por parte de su familia, lo que a su vez podría generar sentimientos de soledad, vacío, tristeza y ansiedad, además de trastornos mentales, afectivos y consumo de drogas.

La información existente evidencia que en muchos casos el estado de salud y las condiciones de reclusión no son los mejores, especialmente en términos ambientales, de derechos humanos (violación de su protección y la de sus hijos), y de la prestación de servicios de salud (nutrición inadecuada, alto hacinamiento y prevalencia de condiciones antihigiénicas que exacerban la mala salud y la transmisión de enfermedades infecciosas), razón por la cual, la atención en salud se valora principalmente como mala 10,11. Esta información la respaldan los hallazgos de Antonetti 12, quien muestra que entre las reclusas las necesidades físicas se satisfacen peor (56,4%) que las necesidades psicológicas (50,9%) y sociales (50,9%).

Algo similar sucede en Indonesia, donde se encontró que existen una serie de dificultades que agravan los problemas de salud de las mujeres en centros penitenciarios, dentro de las cuales destaca el que los sistemas carcelarios sean predominantemente masculinos; por tanto, se les aplica un enfoque carcelario diseñado para hombres 13. Así mismo, un análisis sobre la percepción de salud de las prisioneras en Grecia mostró que es frecuente un estado de salud deficiente (60,4%), como consecuencia de un incremento del consumo de tabaco (16,6%), junto con el consumo de drogas (7,9%), así como el pobre acceso y la calidad de los servicios de salud (46,5% y 49,5%, respectivamente) 14.

De acuerdo con el Observatorio de Política Criminal de Colombia 15, en el país prevalecen condiciones de reclusión indignas para las personas privadas de la libertad. Como en otros escenarios descritos, tiene lugar la vulneración sistemática de los derechos (como a la alimentación y a la salud), el incumplimiento de las condiciones en las que se desarrollaba el tratamiento penitenciario, el acceso a programas de resocialización, a cupos de educación, al trabajo o la enseñanza. No obstante, dentro de las condiciones que más afectan a la salud de esta población se encuentra el hacinamiento 16.

A partir de la literatura que evidencia las condiciones de vulnerabilidad física, social y ambiental de las mujeres recluidas en centros penitenciarios en un contexto global, y considerando la hipótesis planteada sobre las condiciones de la mujer recluida, la cual se ve afectada por las diferencias en el abordaje de la reclusión entre hombres y mujeres, el presente estudio tuvo como objetivo identificar las condiciones biopsicosociales con enfoque de género de mujeres privadas de la libertad en la Cárcel Distrital de Cartagena (Colombia), en el año 2020.

## MATERIALES Y MÉTODOS

Con el fin de responder al planteamiento del problema, la investigación adopto una metodología cuantitativa, descriptiva de corte transversal, la cual orientó la medición de diversos aspectos, y se llegó a formular tendencias y patrones 17.

La población estuvo representada por 154 mujeres privadas de la libertad en la Cárcel Distrital de Cartagena (Colombia), con una muestra total de 130 participantes, derivada de muestreo a conveniencia, y se cumplió con los siguientes criterios de inclusión: mujeres recluidas en la Cárcel Distrital de Cartagena, que desearan voluntariamente participar en el estudio y que tuvieran una estancia mayor de un mes al momento de aplicar el estudio.

La técnica para la recopilación de la información fue la entrevista, donde se aplicó una encuesta tipo Likert, desarrollada en el espacio educativo del centro penitenciario. El cuestionario evaluó los siguientes aspectos: 1. *aspectos sociodemográficos* de las reclusas; 2. *estado de salud percibido* (cuestionario de salud SF-36) 18, puntualizando función física, rol físico, dolor corporal, salud general, vitalidad, función social, rol emocional y salud mental. La consistencia interna de la escala supera el valor mínimo recomendado (a de Cronbach = 0,7) en todas las escalas, excepto en la función social. Las escalas de rol físico, función física y rol emocional obtienen los mejores resultados de fiabilidad y en la mayoría de las ocasiones superan el valor de a de Cronbach = 0,90; 3. y *condiciones de reclusión;* con a) cuestionario de molestias en prisión, útil para evaluar las demandas diarias que más afectan a las personas durante su estancia en prisión, diseñado por Altamirano 6, con a de Cronbach = 0,811; b) versión reducida de la escala de estrés percibido que estima el grado en que las situaciones de la vida son valoradas como estresantes por las personas, diseñado por Cohen *et al.*^19^, con a de Cronbach = 0,84-0,86.

El análisis de la información se realizó mediante el software SPSS (Statistical Package for the Social Sciences), versión 27 en español. Se realizó un análisis univariado mediante el empleo de la medida de frecuencia absoluta y relativa. Para la presentación de la información se utilizaron figuras y tablas.

El análisis de la información se realizó mediante el *software* SPSS (Statistical Package for the Social Sciences), versión 27 en español. Se realizó un análisis univariado mediante el empleo de la medida de frecuencia absoluta y relativa. Para la presentación de la información se utilizaron figuras y tablas.

Dadas las características del tema, y para obtener una información veraz, se cumplieron las siguientes consideraciones éticas: los participantes del estudio accedieron libremente a responder las preguntas realizadas, para lo cual firmaron un consentimiento informado; la información recolectada cumplió con los criterios de confidencialidad, de manera que se garantizó la protección de la identidad y el anonimato.

El estudio se fundamentó, éticamente, en la Resolución 8430 de 1993 de Colombia, y fue clasificado como una investigación con mínimo riesgo, dado que no se realizó ninguna intervención o modificación intencionada de las variables biológicas, fisiológicas, sicológicas o sociales de los individuos que participaron en el estudio. Sin embargo, se evocaron sentimientos que pudieron perturbar a las entrevistadas, por lo que se contó con la presencia de un profesional en psicología para su abordaje, en caso de ser necesario. El análisis cuantitativo de la información se llevó a cabo mediante el *software* SPSS, versión 22 en español.

## RESULTADOS

Las mujeres recluidas en la Cárcel Distrital de Cartagena se caracterizan, sociodemográficamente, por ser relativamente jóvenes, entre los 30 y los 39 años (28,5%), seguidas de aquellas entre los 20 y los 29 años (26,2%); de formación educativa media (bachiller) (53,8%); de estratos socioeconómicos bajos (nivel 1) (74,6%); de fe religiosa católica (64,6%); y por ser madres (91,5%). Los grados penitenciarios que prevalecen son sindicada (57,7%) y condenada (41,5%).

En cuanto a las condiciones y el estado de salud percibido entre las mujeres se pudo evidenciar que se hallan en un estado saludable. Un aspecto para destacar es el índice de masa corporal (IMC) normal (40%), aunque el 33,1% se encuentra en preobesidad. Otro aspecto positivo fue la realización de citologías periódicas (78,5%). Dentro de los aspectos negativos se encuentran la no realización del autoexamen de mama (56,2%) y el no uso de preservativos durante las relaciones sexuales (60,8%).

**Figura 1:**
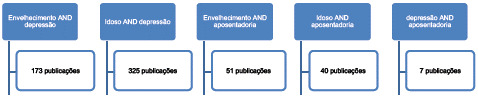
Estado de salud percibido

A pesar de hallarse en un contexto donde predominan unas condiciones de reclusión no tan favorables, la evidencia llegó a concluir que estas mujeres perciben su estado de salud como bueno (48,5%) o regular (37,8%). Algunas de las molestias más frecuentes se asocian con la función social (56,9%), porque la salud física o los problemas emocionales casi siempre han dificultado las actividades sociales (35,4%); la salud mental (45,4%), porque algunas veces se sienten tristes (26,2%) y en otros momentos su motivación es tan baja que nada puede animarlas (26,9%); y por último, afecta su vitalidad (45,4%), producto del cansancio (29,2%) y el agotamiento (24,6%) (Figuras 1 y 2).

Fuente: Encuestas a reclusas de la Cárcel Distrital de Cartagena.

**Figura 2:**
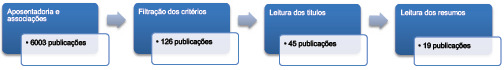
Estado de salud percibido

Fuente: Encuestas a reclusas de la Cárcel Distrital de Cartagena.

Las condiciones ambientales percibida por las mujeres privadas de la libertad se distinguieron por ser buenas en su mayoría. El abordaje por dimensiones llevó a concluir que experimentan molestias con bastante frecuencia (41,5%). Aunque se identificaron factores que generan "demasiada" molestia, sus proporciones no fueron notablemente elevadas, sin que ello les reste gravedad. En términos de las opiniones de las reclusas, les incomoda el ruido (26,9%), el hacinamiento (23,1%), la higiene en general (22,3%), el tipo de personas que hay en la cárcel, la falta de control, las presiones y la violencia (21,5%) (Tabla 1).

**Tabla 1:** . Nivel de molestia que experimentan las reclusas ante diversos aspectos

Respuesta		AA	BB	CC	DD	EE	Total	AA (%)	BB (%)	CC (%)	DD (%)	EE (%)	Total (%)
A	Las compañeras de celda	85	15	13	4	13	130	65,4	11,5	10,0	3,1	10,0	100,0
B	El trato de los funcionarios	82	24	11	2	11	130	63,1	18,5	8,5	1,5	8,5	100,0
≤	Las amenazas	75	18	9	2	26	130	57,7	13,8	6,9	1,5	20,0	100,0
D	Los horarios	73	17	17	9	14	130	56,2	13,1	13,1	6,9	10,8	100,0
E	La higiene durante su período menstrual	64	27	9	7	23	130	49,2	20,8	6,9	5,4	17,7	100,0
F	Las condiciones para cambiarse las veces que pueda en su período menstrual	63	25	9	8	25	130	48,5	19,2	6,9	6,2	19,2	100,0
G	El tipo de gente que hay en la cárcel	60	19	10	13	28	130	46,2	14,6	7,7	10,0	21,5	100,0
H	La higiene en general	56	17	15	13	29	130	43,1	13,1	11,5	10,0	22,3	100,0
I	La falta de control	54	17	19	12	28	130	41,5	13,1	14,6	9,2	21,5	100,0
J	El hacinamiento	53	17	14	16	30	130	40,8	13,1	10,8	12,3	23,1	100,0
K	Las presiones	53	16	21	12	28	130	40,8	12,3	16,2	9,2	21,5	100,0
L	La violencia	50	23	11	18	28	130	38,5	17,7	8,5	13,8	21,5	100,0
M	La comida (calidad, variedad, cantidad)	49	23	22	14	22	130	37,7	17,7	16,9	10,8	16,9	100,0
N	El ruido	47	17	10	21	35	130	36,2	13,1	7,7	16,2	26,9	100,0

AA: Nada; BB: Muy poco; CC: Algo DD: Bastante; EE: Demasiado. Fuente: Encuestas a reclusas de la Cárcel Distrital de Cartagena.

Con relación al estrés, se partió de concebirlo como aquel estado de cansancio mental ocasionado por la exigencia de un rendimiento muy superior al normal, que deviene en alteraciones sobre el organismo que lo experimenta. En este orden de ideas, la información empírica recolectada mostró que las reclusas sienten estrés (50%), mientras que otras lo sienten regularmente (28,5%).

Entre de los elementos que a menudo producen sensación de estrés entre las reclusas se encontraron los siguientes: haber estado segura sobre su capacidad para manejar sus problemas personales (37,7%); haber estado enfadada porque las cosas que le han ocurrido estaban fuera de su control (35,4%); haberse sentido nerviosa o estresada (28,5%); haber sentido que las dificultades se acumulan tanto que no puede superarlas (28,5%), y haber podido controlar las dificultades de su vida (28,5%).

## DISCUSIÓN

El desarrollo del estudio tuvo como aliciente principal exponer los vacíos de la literatura en cuanto a las condiciones de reclusión que experimentan mujeres privadas de la libertad. La falta de interés por analizar las problemáticas de esta población puede deberse a que las mujeres tras las rejas representan, comparativamente con los varones, una proporción relativamente reducida. En este sentido, el Instituto Nacional Penitenciario y Carcelario (Inpec) 20 señala que en el país existe un 93,4% (111 125) de varones encarcelados, mientras que las mujeres representan el 6,6% (7 800). Tal falta de interés, como lo indican Alves *et al.*^21^, hace que se convierta en un grupo marginado y vulnerable.

En conexión con la opinión de Crissman *et al.*^22^, cuando se presta mayor atención y se atienden las necesidades de las reclusas pueden lograrse mejoras en su salud física y mental, en el marco de las garantías a los derechos humanos; además, cuando cumplen la condena y regresan a la comunidad, es factible que se reduzcan las posibilidades de reincidencia, y con ello la comprensión de sus condiciones demográficas, biológicas y ambientales se convierte en un objetivo importante de salud pública en general.

La evidencia empírica arrojó, desde el punto de vista sociodemográfico, que las mujeres privadas de la libertad se caracterizaron, sobre todo, por tener edades entre 30 y 39 años (28,5%), aunque se identificó que había mujeres desde los 19 años hasta mayores de 59. Las reclusas son católicas (64,6%), con hijos (91,5%), provienen de estratos muy bajos (nivel 1: 74,6%) y su escolaridad fue media (bachilleres: 53,8%). Esto rasgos coindicen en gran medida con aquellos dados a conocer en el estudio De Souza y Peixoto 23, quienes detectaron que las mujeres se caracterizan por tener una edad promedio de 30 años, ser pobres, negras y pardas (70,5%), así como presentar un bajo nivel de escolaridad (únicamente 1,5% tienen educación superior).

Desde el punto de vista biológico, se registraron prevalencias algo reducidas de enfermedades, entre ellas las de índole renal (6,9%), artritis (6,2%), enfermedades cardiacas (4,6%), artrosis (3,1%), afecciones vasculares (3,1%) y osteoporosis (1,5%). En este sentido, se evidenciaron diferencias con respecto al trabajo de Mignon 24, pues en las cárceles estadounidenses el 57% de las reclusas estatales y el 52% de las reclusas federales informaron que padecían al menos un problema médico, entre los que se destacaron la artritis, el asma y la hipertensión. De igual forma, Sosa 25 expone un panorama caracterizado por las enfermedades crónicas (66%), tales como la diabetes (19,1%), la hipertensión arterial (12,8%), la gastritis (12,8%) y el asma bronquial (10,6%).

Por otro lado, el consumo de alcohol, drogas y tabaco resultó reducido entre las mujeres estudiadas, con proporciones respectivas de 19,2%, 16,9% y 14,6%. Dicha información contrasta con la expuesta por Ahmed *et al.*^26^, pues el 76% de las reclusas canadienses reconoce tener adicción a las drogas o al alcohol.

El estado de salud percibido por las reclusas fue entre regular (37,8%) y bueno (48,5%). Esto difiere de lo expuesto en revisiones sistemáticas, como la de Ferreira 27, quien concluye que en diversos contextos prevalece el mal estado de salud de las mujeres encarceladas, y que incluso el estado de salud que informan tiende a ser peor, comparativamente, tanto con los reclusos varones como con mujeres no encarceladas.

En el caso de las condiciones ambientales, se consideraron tres dimensiones: molestias, estrés y relaciones interpersonales. En términos de las molestias, hubo un resultado global que apuntó a que se perciben muy pocas (41,5%), u ocasionalmente (32,3%). Dentro de los aspectos evaluados estuvo la comida -en cuanto a calidad, variedad y cantidad-, frente a lo cual, y contrariamente a lo esperado, muchas mujeres indicaron que en nada les molesta (37,7%), y fueron pocas las que mencionaron que las molestaba bastante (10,8%) o demasiado (16,9%). Esto es opuesto a lo detectado por Antonetti *et al.*^12^, dado que en las prisiones italianas las mujeres expresan incapacidad para satisfacer sus preferencias alimentarias y dificultad en el respeto a los requisitos alimentarios.

Igualmente, Van Hout y Mhlanga 28 mencionan algunos países africanos (Zambia, Malawi, Mozambique, Namibia, Camerún, Liberia, Burundi, Lesoto, Chad, Nigeria y Eritrea) en los cuales la falta de raciones suficientes de alimentos, junto con su baja calidad, es lo más común. Con respecto a la investigación de Altamirano 6, también hubo diferencias, en el sentido de que existen molestias en la prisión, básicamente con respecto a la convivencia, que son las más críticas. Aquí se incluyen las amenazas, la violencia, las presiones, la falta de control, la higiene y el ruido.

Frente al estrés, se halló que las encuestadas lo experimentan a menudo (50%), situación que concuerda con la dada a conocer por Constantino *et al.*^29^, quienes calculan que más de la mitad de las reclusas del estado de Río de Janeiro (57,9%) presentan afecciones de este tipo. Un aspecto que se debe destacar, y que es reconocido por Sygit *et al.*^30^, consiste en que la estancia en prisión, al representar un punto de quiebre y un colapso en las relaciones familiares, implica una desmoralización intensificada y un impacto muy fuerte en el modo de afrontar el aislamiento, lo que repercute en la generación de estrés. Estos planteamientos pueden tener manifestación en la evidencia empírica expuesta, puntualmente en el hecho de que, ante la sensación de que todo las controlaba, algunas de estas mujeres reportaron que a menudo experimentan estrés (14,6%), o que lo experimentan muy a menudo (13,8%). Otras, por su parte, expresan haber sentido que las cosas les van bien, que nunca sienten estrés (14,6%), o que casi nunca (16,2%) lo sienten, sumado a aquellas que muy a menudo consideran haber sentido que las dificultades se acumulan tanto que no pueden superarlas (28,5%).

Las relaciones interpersonales fueron entre excelentes (48,5%) y regulares (46,9%). En efecto, y de modo desagregado, por un lado, las relaciones fueron excelentes con los guardias y el personal de la cárcel (44,6% y 43,1%, respectivamente), pero para otro segmento de las reclusas, con dichos sujetos las relaciones fueron más bien regulares (43,1% y 44,6%, de modo respectivo). Además, con las compañeras las relaciones suelen ser entre regulares (45,4%), buenas (20%) y excelentes (33,1%). Esto, en cierto modo, es similar a lo expuesto por García y Melendro 31, en la medida en que en las prisiones femeninas españolas la gran mayoría de las reclusas (80%) no han tenido problemas en sus relaciones interpersonales con el personal de la prisión; adicionalmente, las relaciones entre compañeras han sido catalogadas como positivas (80,5%).

El estudio permitió evidenciar necesidades en salud que demanda la población penitenciaria de la Cárcel Distrital de Cartagena, entre ellas, la falta de conocimiento en relación con la salud sexual y reproductiva, lo que se convierte en un agravante a nivel social por el número de embarazo en las mujeres recluidas; también se evidenciaron consecuencias para la salud que conllevan la obesidad y el sedentarismo, lo que predispone al desarrollo de enfermedades cardiovasculares. Así mismo, es preciso fortalecer la salud mental, específicamente asociada a la reconciliación con la sociedad y la autoestima; todo de la mano de la religión, la familia y la sana convivencia, fortalezas identificadas por las reclusas. Cabe resaltar la importancia de seguir trabajando en sus habilidades laborales para una inserción social provechosa, además de incluir en estos proyectos un enfoque empresarial mediante la educación superior.

El contexto antes mencionado conlleva la necesidad de próximas investigaciones de naturaleza cualitativa que permitan comprender mejor las condiciones de dicha población, especialmente aquellas investigaciones dirigidas a develar la percepción de las mujeres privadas de la libertad frente a los factores psicosociales que incidieron en su reclusión, así como los efectos de la reclusión una vez recobrada la libertad. Esto para que, por medio de sus vivencias, aporten a las políticas públicas en pro del restablecimiento de sus necesidades primarias, la reconciliación y la inclusión social ♠
